# Supplementary material for: Patterns of Intron Gain and Loss in Fungi
Source: PLoS Biol. 2004 Nov 30;2(12):e422. doi: 10.1371/journal.pbio.0020422 (PMC532390; doi:10.1371/journal.pbio.0020422)
Supplement: Table S1 — Also available at http://genes.mit.edu/NielsenEtAl/. (4.3 MB ZIP). [file pbio.0020422.st001.zip › NielsenEtAl/html/988.html]

AN4323.1.NCU04754.1.MG02489.1.FG09512.1


```
 CLUSTAL W (1.82) Multiple Sequence Alignments - Introns Inserted


Sequence 1: NCU04754.1	427 aa
Sequence 2: MG02489.1	404 aa
Sequence 3: FG09512.1	408 aa
Sequence 4: AN4323.1	495 aa
Alignment Length: 518 aa
Number Identitical Residues: 235 aa
Alignment Score (without introns) 11007


MG02489.1 	MAPSMLR----SILRSSPSAARRWSRHYSVKAEVASTAQPK----DIEPAQLKITKTSKP
NCU04754.1	MFRTSLR----RAAAFQPARPCFGAFSATAARQYSAATGTA----DINPSKLKVEKTTQP
FG09512.1 	MMRPMLARSALRSARLSSFRPLCQAQRFSIKAEAASSAQHM----GLDASKLTIQKTGNH
AN4323.1  	-MKSLHQLARPRALPSFAAPRAQSARSNRLWQRCFSATRAASEGAQLDPSKLTITKTSTP
          	   .    :        .      :       .  :::   :..: ::.::*.: **   

MG02489.1 	GVLDKPENLLFGRKFS----------------------D1HMITCEWEQTNGWQQPQIVP
NCU04754.1	KGLLKPEELVFGRNFTGTVQNLPSKNAQSALTNFRATPD~HMLTIEWTKENGWNAPEIKP
FG09512.1 	KTLSKPETLVFGKEFT----------------------D1HMLAIEWNQDKGWLEPKITP
AN4323.1  	KELLPAKDLVFGKNFT----------------------D1HMFTVEWTAKDGWLAPQIVP
          	  *  .: *:**::*:                      * **:: **   .**  *:* *

MG02489.1 	YQNLSMDPATCVLHYAFTCFEGMKAYKDKSGQVRLFRPDKNMERFNKSAARIALPNFSSP
NCU04754.1	YQNLSLDPATCVFHYAFECFEGMKAYKDKAGKIRLFRPDKNMARFNKSAARIALPTFDSP
FG09512.1 	YQNLSLDPATCVFHYAFECFEGMKAYKDKNGKVRLFRPDMNMARLNKSAARIALPTFEPA
AN4323.1  	YQKLQLDPSACVFHYAFECFEGMKAYKDSKGQIRLFRPDKNMQRLNKSSARIALPTVDGE
          	**:*.:**::**:**** **********. *::****** ** *:***:******...  

MG02489.1 	ALIDIISQYAKLESRFIPD~QRGFSLYLRPTMIGTQKTLGVSPPGSAMLFVIASPVGPYY
NCU04754.1	ALIDLIAKLVKLDSRFIPE~QRGYSLYLRPTMIGTQKTLGVGPPGSALLYVIASPVGPYY
FG09512.1 	EFAQLISKLVNLDSRFIPD~QRGYSLYLRPTMIGTQKTLGVGPPGSALLYCIASPVGPYY
AN4323.1  	ALTKLIGELVKLDSRFIPD2ARGYSLYLRPTMIGTQSTLGVGPPGSALLFVIASPVGPYY
          	 : .:*.: .:*:*****:  **:************.****.*****:*: *********

MG02489.1 	PTGFKAVSLEATDYAVRAWPGGAGDKKLGANYAPCIVPQREAMARGFQQNLWLFGEEEYV
NCU04754.1	PTGFKAVSLEATDYAVRAWPGGVGDKKLGANYAPCIVPQVEAMSRGFQQNLWLFGEEEYV
FG09512.1 	PTGFKAVSLEATDYAVRAWPGGVGDKKLGANYAPCILPQLQAASRGFQQNLWLFGEEEFV
AN4323.1  	PTGFKAISLEATDYAVRAWPGGVGDKKLGANYAPCIVPQLEAASRGFQQNLWLFGEEEYV
          	******:***************.*************:** :* :**************:*

MG02489.1 	TEVGTMNMFVAIKNKQTGQKELLTAPLDGTILEGVTRDSILALARERLVPEGWAVSERKF
NCU04754.1	TEVGTMNFFVAIKDKKTGQKTLVTAPLDGTILEGVTRDSVLALAREKLAPEGWKIEERKF
FG09512.1 	TEVGTMNMFVALKNKETGQKELITAPLDGTILEGVTRDSVLALARERLIPQGWKITERKY
AN4323.1  	TEVGTMNLFIALKNKETGKKELVTANLDGTILEGVTRDSVLALARERLVPKGWQVSERKI
          	*******:*:*:*:*:**:* *:** *************:******:* *:** : *** 

MG02489.1 	TMKELHEASTEGRLIEAFGSGTAAIVSPVRSIAWKGKLVHCGLQDHEESGEIAMRMKGWI
NCU04754.1	TMKELAEAANEGRLIEAFGAGTAAIVSPVRSISWKGQLVNCGLKPEEESGELTMQVKNWM
FG09512.1 	TMKELAEAADEGRLLEAFGTGTAAIVSPVRSISWKGKLVDCGLSEVEESGEIALQMKNWI
AN4323.1  	RMAEVAEAADEGRLLEVFGAGTAAIVSPVRTISYRGRLVNCGLKETEEAGEIASQMKNWI
          	 * *: **: ****:*.**:**********:*:::*:**.***.  **:**:: ::*.*:

MG02489.1 	ESIQYGDEDHEWS~HV~C------------------------------------------
NCU04754.1	EARQYGDEEHEWS~YV~CE-----------------------------------------
FG09512.1 	EAIQYGDEEHEWS~YT~I------------------------------------------
AN4323.1  	EGIQYGDEEHPWR2II0YWQSIRFIFNSNPFLQSSLSDGRALVREKNYPTYLYSAPRVSS
          	*. *****:* *       .:     .:..  .:: :.. :   ... .:   ::.  ::

MG02489.1 	------------------------------------------
NCU04754.1	------------------------------------------
FG09512.1 	------------------------------------------
AN4323.1  	GFHQTPAHIIPCIIHLVEAELNQAQNMNPCQAVNKPALRDNQ
          	.  .:.:   ..     .:. ..:.. ....: ...:  ...
```
